# Moxibustion therapy for chronic spontaneous urticaria: A protocol for systematic review and meta-analysis

**DOI:** 10.1097/MD.0000000000023226

**Published:** 2020-11-13

**Authors:** Sijia Shen, Meiling Wang, Jingcheng Dong

**Affiliations:** a Huashan Hospital Affiliated to Fudan University; b 905 Hospital of People's Liberation Army Navy, Shanghai, People's Republic of China.

**Keywords:** chronic spontaneous urticaria, moxibustion therapy, protocol, systematic review

## Abstract

**Background:**

Chronic spontaneous urticaria (CSU) is a common disease in clinical, and often recrudescent. However, sometimes Western medicine treatments such as antihistamines cannot completely control the symptoms of CSU; therefore, more effective and optimized treatments are needed. Numerous studies have confirmed that moxibustion therapy is effective in treating CSU. Given that no relevant systematic reviews and meta-analysis have been carried out, we set out to prove the effect of moxibustion therapy for CSU.

**Methods:**

This protocol will be conducted based on the PRISMA-P guidelines and comply with the recommendations of the Cochrane Collaboration Handbook for Systematic Reviews. We plan to search the subsequent databases: PubMed, Web of Science, EMBASE.com and Cochrane Library, China National Knowledge Infrastructure, WanFang Database, Chinese Science Journal Database, and China Biomedical Literature Database. The studies will be screened under the eligibility criterion. The quality of the studies will be assessed based on the Cochrane risk bias tool. Ultimately, Review Manager 5.3 will be used for statistical analysis.

**Results:**

This research will comprehensively evaluate the effectiveness of moxibustion therapy for CSU, and provide a more reasonable and effective treatment plan for CUS.

**Conclusion:**

This research will bring new evidence for the efficacy of moxibustion therapy in the treatment of CSU and provide a basis for future clinical applications.

**Inplasy registration number:**

INPLASY2020100045

## 1 Introduction

Urticaria is a localized edema response caused by the expansion of cutaneous small blood vessels and mucous membranes and increased permeability with the activation of mast cells as the core.^[1–3]^ It is often accompanied by wind masses of varying sizes, itching, and angioedema. According to the statistics of the World Allergy Organization, about 20% of people have had urticaria during their lifetime.^[4]^ The course of chronic urticaria is more than 6 weeks. There are different types of chronic urticaria, usually divided into spontaneous and inducible urticaria.^[5,6]^ Among them, chronic spontaneous urticaria (CSU) is the most common clinical type of chronic urticaria.^[7]^ CSU is marked by severe itching, often recrudescent, and the course is often long and unpredictable. The pathogenesis of CSU is complex, and it is often difficult to find a clear cause.^[8–10]^ Therefore, it is difficult to carry out targeted treatment of the cause, and only symptomatic treatment is currently available.^[11,12]^ However, sometimes symptomatic treatment such as antihistamines cannot completely control the symptoms of CSU,^[13,14]^ it will lead to a serious influence on the quality of life of patients,^[15]^ and also greatly increase the pressure on public health.^[16,17]^ Therefore, more effective and optimized treatments are required.

Moxibustion therapy is a method of curing diseases by burning traditional Chinese medicine-wormwood (artemisia argyi) on or nearby human acupoints.^[18]^ Wormwood has the effect of warming meridians and dispelling cold, and can dispel dampness and relieve itching used externally.^[19]^ According to modern mechanism studies, moxibustion therapy has antiallergic effects and can treat a variety of diseases related to allergic reactions, including urticaria, allergic rhinitis, irritable bowel syndrome,^[20–22]^ etc. Moxibustion therapy also has antibacterial effects, which can inhibit various bacteria such as gram-negative bacteria, *Streptococcus beta*, *Pseudomonas aeruginosa*, etc.^[23,24]^ Besides, moxibustion therapy is effective in treating tumors and digestive system diseases, has certain effects on diseases of the nervous system and musculoskeletal, and also can regulate immunity system.^[25–28]^ Numerous studies have confirmed that moxibustion therapy is effective in treating CSU.^[20,29]^ It can relieve the symptoms of itching, reduce the frequency of CSU, and finally improve quality of life. Given no relevant systematic reviews and meta-analysis has been conducted, we set out to prove the effect of moxibustion therapy for CSU.

## 2 Methods

### 2.1 Study registration

This protocol will be conducted based on the PRISMA-P guidelines^[30]^ and abide by the recommendations of the Cochrane Collaboration Handbook for Systematic Reviews.^[31]^ INPLASY registration number is INPLASY2020100045.

### 2.2 Database

Regarding randomized controlled trials (RCTs) to be included, we plan to search the subsequent databases: PubMed, Web of Science, EMBASE.com and Cochrane Library, China National Knowledge Infrastructure (CNKI), WanFang Database, Chinese Science Journal Database (VIP), and China Biomedical Literature Database (CBM). The publications reported in Chinese and English will be included, until July 31, 2020. Besides, references will be manually searched for relevant studies to find studies that may qualify.

### 2.3 Search strategy

The following items are the key search criteria: (“urticaria” OR “hives” OR “dermographism” OR “nettle rash”) AND (“moxibustion”) AND (“randomized”). This search strategy will be modified for different database requirements. Table 1 shows a search strategy for the PubMed database.

**Table 1:** Search strategy for PubMed database.

Number	Search items
1	urticaria.MeSH
2	urticarias
3	hives
4	dermographism
5	nettle rash
6	rubella
7	roteln
8	wheal
9	or 1–8
10	moxibustion
11	needle warming moxibustion
12	thunder fire moxibustion
13	mild moxibustion
14	taiyi moxibustion
15	suspended moxibustion
16	or 10–15
17	randomized controlled trial
18	controlled clinical trial
19	controlled trial
20	randomised
21	random
22	randomization
23	randomly
24	placebo
25	trial
26	group
27	or 17–26
28	9 and 16 and 27

### 2.4 Study inclusion and exclusion criteria

#### 2.4.1 Types of study

This systematic review will clarify whether moxibustion therapy is effective for CSU? This paper will comprehensively collect RCTs and only include high-quality RCTs after screening.

We will only include publications in which languages are limited in Chinese and English, while other languages will be excluded. Besides, follow-up will not be restricted. The papers which unable to find the full article will be excluded. Controlled (nonrandomized) clinical trials, cohort studies, nonhuman studies, non-RCTs, case reports, observational study, random crossover studies, retrospective studies, single-arm studies, and reviews will be excluded.

#### 2.4.2 Types of participants

Despite citizenship, gender, race, as for age, the patients required to be included are between 18 and 65 years old.

CSU must be diagnosed according to the following international or national diagnostic criteria. The international standard refers to “2007 Guidelines for evaluation and management of urticaria in adults and children by British Association of Dermatologists.” The domestic standard refers to “Skins and Venereology,” “Clinical Dermatology,” “Chinese Clinical Dermatology,” “Urticaria diagnosis and treatment guide” stipulated by the Chinese Medical Association, 2007.

#### 2.4.3 Types of intervention

The intervention group must use moxibustion therapy, including needle warming moxibustion, thunder fire moxibustion, mild moxibustion, taiyi moxibustion, and suspended moxibustion.

The control interventions of the following processing will be included:

1. Moxibustion therapy is compared with other active therapies.
2. Moxibustion therapy is compared with sham therapies or placebo.
3. Moxibustion therapy in addition to active therapy compared with the same active therapy.
4. No treatment in the control group.

Any treatment related to moxibustion therapy in the control group will be excluded.^[32]^

#### 2.4.4 Types of outcomes

The main outcomes are based on the Urticarial activity score and the European MILOR rating scale. The additional outcomes include: Assessment of efficacy: symptom score reduce index and Global symptom improvement, assessment of quality of life: Dermatology Life Quality Index, Assessment of Objective index: IgE.

### 2.5 Data process and analysis

#### 2.5.1 Screening of studies

**Figure 1:**
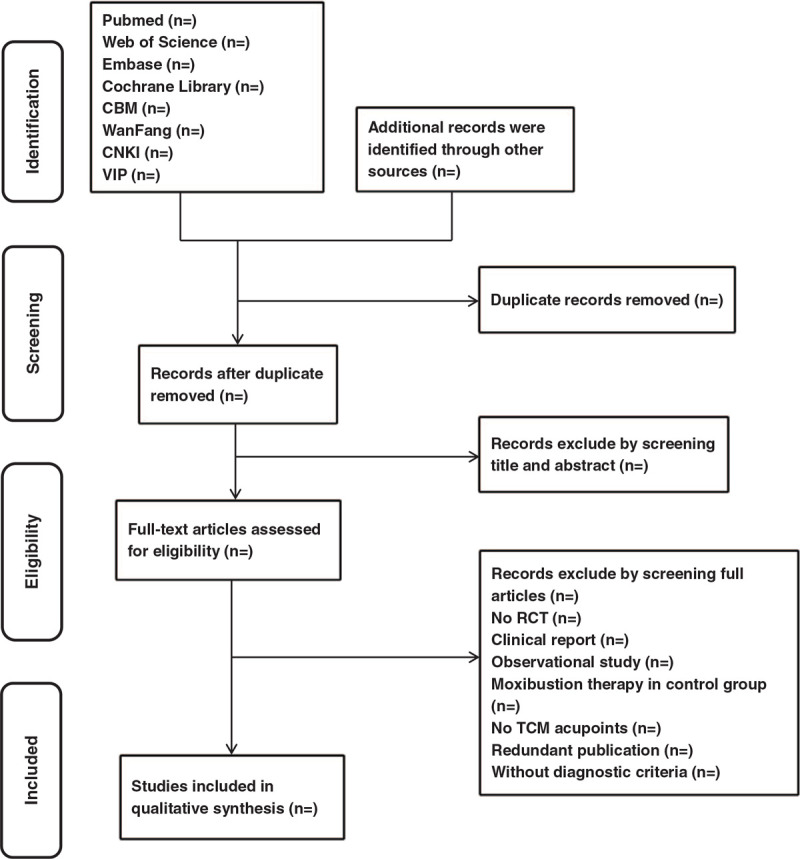
PRISMA flow chart of the literature screening and selection process.

Two reviewers will independently browse the titles and abstracts of the papers, read the full text if necessary, and screen the papers based on relevant inclusion criteria such as outcome indicators and diagnostic criteria. If there is a disagreement, the 2 reviewers will consult a third party of a clinically experienced reviewer. Figure 1 shows the study screening and primary selection process flow chart based on PRISMA.

#### 2.5.2 Data extraction

We will extract data on basic information and primary information. The basic information will include the title of the paper, author, publication year, random method, allocation concealment, blind method, etc. The primary information will include intervention measures, the mean age of the participants, the number of included cases, and effective cases in the therapy group, outcomes, etc. If the demanded data is not published in the paper, we will approach the initial author by email for the related data.

#### 2.5.3 Assessment of risk of bias

All of the methodological qualities of the trials will be inspected and evaluated by 2 authors (SS and MW) independently. If the relevant data, such as blinding methods or random methods, is not published in the paper, we will approach the initial author by telephone or email for the related data. The Cochrane Collaboration's bias risk assessment tool will be used by the 2 authors to evaluate the bias risk of the subsequent areas of all included studies: random sequence, assignment sequence concealment, incomplete outcome data, blinding of trial personnel and participants, selective outcome reporting, blinding of outcome assessors, and other biases. If there is a disagreement between the 2 reviewers, they will consult a third clinically experienced reviewer (JD).

### 2.6 Summary statistics

The Review Manager 5.3 will be used for statistical analysis. Select according to the type of data, if the data is binary, use the odds ratio; if the data is continuous, use standardized mean difference.^[33]^

#### 2.6.1 Assessment of heterogeneity

Choose the fixed or random effect model under the heterogeneity test results of the study. If *P*>.10 and I^2^≤50%, the heterogeneity is considered satisfactory, and the fixed effect model is used; if *P*≤.10 and I^2^>50%, the heterogeneity is considered to be out of the acceptable range, and the random-effects model is used to analyze the reasons for the heterogeneity.^[34,35]^

#### 2.6.2 Sensitivity analysis

When sufficient researches are included, a sensitivity analysis of the main outcomes will be carried out. If the sensitivity analysis does not substantially change the results, the results are credible; if the sensitivity analysis yields different conclusions, suggesting that there are potentially important factors that affect the effects of interventions. It indicates should be cautious when interpreting the results and drawing conclusions, and the factors need to be confirmed later.

#### 2.6.3 Assessment of reporting biases

In case of more than 10 studies all contain a certain outcome index, the Revman 5.3 software will be used to draw a funnel chart for publication bias analysis. If the scatter points on the funnel chart show a symmetrical distribution, it can be considered that the included literature has no publication bias; if the funnel chart is asymmetry or incomplete indicates that there is a higher possibility of publication bias in the included literature.

#### 2.6.4 Subgroup analysis

No subgroup plan in this review previously.

## 3 Discussion

CUS is a common allergic disease in clinical. The incidence of urticaria is about 20% during a person's lifetime.^[4]^ There is a study that indicated^[36]^ the incidence of chronic urticaria in European and American populations is 0.1% to 3%. CUS not only leads to a serious influence on the quality of life of patients,^[16,37]^ but is also related to high costs. Its diagnosis and treatment greatly increase the pressure on public health.^[38,39]^ Numerous studies have confirmed that moxibustion therapy is effective in treating CSU.^[20,29]^ Given that no relevant systematic reviews and meta-analysis have been carried out, we set out to prove the effect of moxibustion therapy for CSU, to provide a more reasonable and effective treatment plan for CUS.

## Author contributions

**Conceptualization:** Sijia Shen.

**Methodology:** Sijia Shen.

**Supervision:** Jingcheng Dong.

**Validation:** Meiling Wang.

**Writing – original draft:** Sijia Shen, Meiling Wang.

## References

[1] Seirin-Lee S Yanase Y Takahagi S A single reaction-diffusion equation for the multifarious eruptions of urticaria. PLoS Comput Biol 2020;16:e1007590. 31940345 10.1371/journal.pcbi.1007590 PMC6961880

[2] Church MK Kolkhir P Metz M The role and relevance of mast cells in urticaria. Immunol Rev 2018;282:232–47. 29431202 10.1111/imr.12632

[3] Mac Glashan DW Jr Ig E-dependent signaling as a therapeutic target for allergies. Trends Pharmacol Sci 2012;33:502–9. 22749712 10.1016/j.tips.2012.06.002 PMC3427396

[4] Zuberbier T Aberer W Asero R The EAACI/GA2LEN/EDF/WAO guideline for the definition, classification, diagnosis and management of urticaria. Allergy 2018;73:1393–414. 29336054 10.1111/all.13397

[5] Skander D Allenova A Maurer M Omalizumab is effective in patients with chronic spontaneous urticaria plus multiple chronic inducible urticaria. Eur Ann Allergy Clin Immunol 2020;Online ahead of print. 10.23822/EurAnnACI.1764-1489.153 32496030

[6] Dortas Junior S Azizi G Valle S Efficacy of omalizumab in chronic spontaneous urticaria associated with chronic inducible urticaria. Ann Allergy Asthma Immunol 2020;S1081-1206(20)30406-3. 10.1016/j.anai.2020.06.011 32544531

[7] Balp MM Weller K Carboni V Prevalence and clinical characteristics of chronic spontaneous urticaria in pediatric patients. Pediatr Allergy Immunol 2018;29:630–6. 29679413 10.1111/pai.12910

[8] Kaplan AP Diagnosis, pathogenesis, and treatment of chronic spontaneous urticaria. Allergy Asthma Proc 2018;39:184–90. 29669665 10.2500/aap.2018.39.4121

[9] Maurer M Eyerich K Eyerich S Urticaria: Collegium Internationale Allergologicum (CIA) Update 2020. Int Arch Allergy Immunol 2020;181:321–33. 32224621 10.1159/000507218 PMC7265766

[10] Pier J Bingemann TA Urticaria, angioedema, and anaphylaxis. Pediatr Rev 2020;41:283–92. 32482691 10.1542/pir.2019-0056

[11] Sharma M Bennett C Cohen SN H1-antihistamines for chronic spontaneous urticaria. Cochrane Database Syst Rev 2014;2014:CD006137. 10.1002/14651858.CD006137.pub2 PMC6481497 25397904

[12] Fedorowicz Z van Zuuren EJ Hu N Histamine H2-receptor antagonists for urticaria. Cochrane Database Syst Rev 2012;2012:CD008596. 10.1002/14651858.CD008596.pub2 PMC7390502 22419335

[13] Orden RA Timble H Saini SS Efficacy and safety of sulfasalazine in patients with chronic idiopathic urticaria. Ann Allergy Asthma Immunol 2014;112:64–70. 24331396 10.1016/j.anai.2013.09.028

[14] Choi JH Lee DH Song WJ The KAAACI/KDA evidence-based practice guidelines for chronic spontaneous urticaria in Korean Adults and Children: Part 2. Management of H1-Antihistamine-Refractory Chronic Urticaria. Allergy Asthma Immunol Res 2020;12:750–70. 32638557 10.4168/aair.2020.12.5.750 PMC7346997

[15] Weldon DR Quality of life in patients with urticaria. Allergy Asthma Proc 2006;27:96–9. 16724624

[16] Balp MM Khalil S Tian H Burden of chronic urticaria relative to psoriasis in five European countries. J Eur Acad Dermatol Venereol 2018;32:282–90. 28898460 10.1111/jdv.14584 PMC6084337

[17] Caballero T Prior N Burden of illness and quality-of-life measures in angioedema conditions. Immunol Allergy Clin North Am 2017;37:597–616. 28687112 10.1016/j.iac.2017.04.005

[18] Liu M Zhu J Wu S De novo assembly and analysis of the Artemisia argyi transcriptome and identification of genes involved in terpenoid biosynthesis. Sci Rep 2018;8:5824. 29643397 10.1038/s41598-018-24201-9 PMC5895812

[19] Yun Y Shin S Kim KS Three cases of cutaneous warts treated with moxibustion. Explore (NY) 2016;12:277–81. 27234466 10.1016/j.explore.2016.04.003

[20] Shi Y Zhou S Zheng Q Systematic reviews of pharmacological and nonpharmacological treatments for patients with chronic urticaria: an umbrella systematic review. Medicine (Baltimore) 2019;98:e15711. 31096521 10.1097/MD.0000000000015711 PMC6531058

[21] Shiue HS Lee YS Tsai CN Treatment of allergic rhinitis with acupoint herbal plaster: an oligonucleotide chip analysis. BMC Complement Altern Med 2016;16:436. 27814709 10.1186/s12906-016-1418-0 PMC5097372

[22] Zhao JM Chen L Zhou CL Comparison of electroacupuncture and moxibustion for relieving visceral hypersensitivity in rats with constipation-predominant irritable bowel syndrome. Evid Based Complement Alternat Med 2016;2016:9410505. 27738447 10.1155/2016/9410505 PMC5055954

[23] Wei Q Bhasme P Wang Z Chinese medicinal herb extract inhibits PQS-mediated quorum sensing system in Pseudomonas aeruginosa. J Ethnopharmacol 2020;248:112272. 31586695 10.1016/j.jep.2019.112272

[24] Wang H Zhang M Ma Y Selective inactivation of Gram-negative bacteria by carbon dots derived from natural biomass: Artemisia argyi leaves. J Mater Chem B 2020;8:2666–72. 32142085 10.1039/c9tb02735a

[25] Yang FM Yao L Wang SJ Current tracking on effectiveness and mechanisms of acupuncture therapy: a literature review of high-quality studies. Chin J Integr Med 2020;26:310–20. 30707414 10.1007/s11655-019-3150-3

[26] Chen Z Zhou D Wang Y Fire needle acupuncture or moxibustion for chronic plaque psoriasis: study protocol for a randomized controlled trial. Trials 2019;20:674. 31801593 10.1186/s13063-019-3736-2 PMC6894135

[27] Bae K Kim E Kong JS Integrative cancer treatment may have a survival benefit in patients with lung cancer: a retrospective cohort study from an integrative cancer center in Korea. Medicine (Baltimore) 2019;98:e16048. 31261510 10.1097/MD.0000000000016048 PMC6617466

[28] Zimmermann-Klemd AM Reinhardt JK Morath A Immunosuppressive Activity of <i>Artemisia argyi</i> Extract and isolated compounds. Front Pharmacol 2020;11:402. 32322200 10.3389/fphar.2020.00402 PMC7157444

[29] Ma C Sivamani RK Acupuncture as a treatment modality in dermatology: a systematic review. J Altern Complement Med 2015;21:520–9. 26115180 10.1089/acm.2014.0274

[30] Moher D Stewart L Shekelle P Implementing PRISMA-P: recommendations for prospective authors. Syst Rev 2016;5:15. 26822481 10.1186/s13643-016-0191-y PMC4730599

[31] Deeks JJ Higgins JPT Altman DG Cochrane handbook for systematic reviews of interventions version 5.1. 0 (updated March 2011). 2011, The Cochrane Collaboration.

[32] Hon KL Leung AKC Ng WGG Chronic Urticaria: an overview of treatment and recent patents. Recent Pat Inflamm Allergy Drug Discov 2019;13:27–37. 30924425 10.2174/1872213X13666190328164931 PMC6751347

[33] DerSimonian R Kacker R Random-effects model for meta-analysis of clinical trials: an update. Contemp Clin Trials 2007;28:105–14. 16807131 10.1016/j.cct.2006.04.004

[34] Higgins JPT Thompson SG Quantifying heterogeneity in a meta-analysis. Stat Med 2002;21:1539–58. 12111919 10.1002/sim.1186

[35] Higgins JPT Thompson SG Deeks JJ Measuring inconsistency in meta-analyses. BMJ 2003;327:557–60. 12958120 10.1136/bmj.327.7414.557 PMC192859

[36] Kozel MM Sabroe RA Chronic urticaria: aetiology, management and current and future treatment options. Drugs 2004;64:2515–36. 15516152 10.2165/00003495-200464220-00003

[37] Mann C Dreher M Weeß HG Sleep disturbance in patients with urticaria and atopic dermatitis: an underestimated burden. Acta Derm Venereol 2020;100:adv00073. 32016441 10.2340/00015555-3416 PMC9128904

[38] Maurer M Abuzakouk M Bérard F The burden of chronic spontaneous urticaria is substantial: real-world evidence from ASSURE-CSU. Allergy 2017;72:2005–16. 28543019 10.1111/all.13209 PMC5724512

[39] Hoskin B Ortiz B Paknis B Exploring the real-world profile of refractory and non-refractory chronic idiopathic urticaria in the USA: clinical burden and healthcare resource use. Curr Med Res Opin 2019;35:1387–95. 30793986 10.1080/03007995.2019.1586222

